# Bispectral index versus COMFORT score to determine the level of sedation in paediatric intensive care unit patients: a prospective study

**DOI:** 10.1186/cc2977

**Published:** 2004-11-10

**Authors:** Andreas E Triltsch, Grit Nestmann, Helmut Orawa, Maryam Moshirzadeh, Michael Sander, Joachim Große, Arka Genähr, Wolfgang Konertz, Claudia D Spies

**Affiliations:** 1 Department of Anesthesiology and Intensive Care Medicine, Campus Benjamin Franklin, Charité University Hospital Berlin, Berlin, Germany; 2 Professor of Cardiac Surgery, Department of Pediatrics, Campus Benjamin Franklin, Charité University Hospital Berlin, Berlin, Germany; 3 Professor of Anesthesiology, Department of Medical Statistics and Clinical Epidemiology, Campus Benjamin Franklin, Charité University Hospital Berlin, Berlin, Germany

**Keywords:** bispectral index, electroencephalography, intensive care unit, paediatric, sedation

## Abstract

**Introduction:**

Most clinicians give sedatives and analgesics according to their professional experience and the patient's estimated need for sedation. However, this approach is prone to error. Inadequate monitoring of sedation and analgesia may contribute to adverse outcomes and complications. With this in mind, data obtained continuously using nonstimulating methods such as bispectral index (BIS) may have benefits in comparison with clinical monitoring of sedation. The aim of this prospective observational trial was to evaluate the use of electroencephalographic (EEG) BIS for monitoring sedation in paediatric intensive care unit (PICU) patients.

**Methods:**

Forty paediatric patients (<18 years) were sedated for mechanical ventilation in a cardiac surgical and general PICU. In each paediatric patient BIS and COMFORT score were obtained. The study protocol did not influence ongoing PICU therapy. BIS and corresponding COMFORT score were collected three times for each patient. Measurements with the best starting EEG impedances were analyzed further. Deep sedation was defined as a COMFORT score between 8 and 16, and light sedation as a score between 17 and 26. Biometric and physiological data, and Pediatric Risk of Mortality III scores were also recorded.

**Results:**

There was a good correlation (Spearman's rho 0.651; *P *= 0.001) between BIS and COMFORT score in the presence of deep sedation and low starting impedance. Receiver operating characteristic (ROC) analysis revealed best discrimination between deep and light sedation at a BIS level of 83.

**Conclusion:**

In the presence of deep sedation, BIS correlated satisfactorily with COMFORT score results if low EEG impedances were guaranteed.

## Introduction

Most paediatric intensive care unit (ICU) patients need sedative and analgesic drugs during mechanical ventilation [1]. Sedatives and analgesics are given to improve comfort, to reduce pain, to facilitate aggressive ICU therapy (i.e. mechanical ventilation or insertion of intravascular lines) and to avoid accidental removal of medical devices. Most clinicians give sedatives and analgesics according to their professional experience and the patient's estimated need for sedation. Inadequate monitoring of sedation and analgesia may contribute to adverse outcomes and complications [2].

Only a few clinical scores have been validated for estimating the level of sedation in paediatric ICU patients. The best evaluated score is the COMFORT score [3]. The COMFORT score consists of eight categories and can evaluate a child's behaviour and physiological responses to discomfort, fear and pain in approximately 2 min. The COMFORT score is age independent because age-adapted physiological parameters are used. Apart from the examination of muscular tone, calculation of the COMFORT score does not require any stimulation of the patient. The COMFORT score can be divided into three groups. A score of 8–16 points corresponds to deep sedation, 17–26 indicates light sedation and 27–40 indicates inadequate sedation [4]. Common concerns about clinical sedation scales are that the employed parameters are susceptible to subjective interpretation and that information about the level of sedation can only be obtained intermittently. Deeper levels of sedation are difficult to assess using clinical sedation scores. Sufficient information cannot be obtained regarding over-sedation, which is associated with adverse outcomes, prolonged ICU stay and increased costs [5-7]. Additionally, patients treated with muscle relaxants cannot be evaluated using clinical rating scores. In paediatric ICU patients, neuromuscular blocking agents are used in approximately 6–16% of ventilatory support days [8]. A point of criticism regarding the COMFORT score is that physiological parameters such as haemodynamic indices and heart rate, which contribute to the score (Table 1), can be influenced by ICU therapy. Therefore, objective tools with which to measure the level of sedation are urgently needed so that over-sedation can be avoided and the level of sedation adjusted if muscle relaxants are given.

With this in mind, data obtained continuously using nonstimulating methods such as bispectral index (BIS) may have benefits in comparison with clinical monitoring of sedation. BIS is a processed electroencephalographic (EEG) parameter that provides a measure of sedative levels on a relative scale [9-11]. For various agents (e.g. propofol and midazolam) it has been shown that the BIS may correlate with dose-dependent levels of anaesthesia [10,12-14] and ICU sedation [15-18]. Level of sedation and changes in memory function correlated well with BIS in volunteers [19]. The BIS monitor was initially designed to measure the level of consciousness in adults during anaesthesia. In paediatric patients age-specific changes in EEG activity could complicate interpretation of BIS measurements [20]. Nevertheless, the first reports of use of BIS in paediatric anaesthesia were promising [21-23]. Recently, McDermott and coworkers [24] investigated the use of BIS during sedation in children undergoing elective diagnostic or therapeutic procedures. Those investigators found good correlation between BIS and the University of Michigan Sedation Scale. In a paediatric intensive care unit (PICU) setting, four groups found a good to moderate correlation between BIS and clinical scores [25-28]. Unfortunately, those studies used clinical scores that have not been evaluated in paediatric patients [25,27], the investigators were not sufficiently blinded to the BIS results [25,26,28], or uncontrolled repeated measurements in one patient were included in the study [25,27,28].

The objective of the present study was to evaluate use of the BIS to monitor levels of sedation in paediatric ICU patients in a blinded and prospective manner, and to compare it with use of the COMFORT score. To avoid bias by repeating analysis of data from the same patient, each patient was included only once in the statistical analysis. In addition, we wished to focus on the age of the children and on quality of EEG data.

## Methods

Following agreement from the local ethics committee and once informed consent from the legal guardians (parents) had been obtained, we studied 50 paediatric patients admitted to our PICU. All included patients met primary inclusion (age <18 years, requirement for mechanical ventilation) and exclusion criteria (brain trauma, any kind of end-stage disease, use of muscle relaxants or persistent postoperative relaxation according to train-of-four monitoring, intractable agitation). After enrolment patients were excluded if correct attachment of electrodes was impossible, impedances did not comply with the quality requirements of the manufacturer (<10 kΩ, signal quality index >0.8, less than threefold deviation between electrode impedances), or recording could not be repeated at least twice. The sedation and/or analgesic regimens were not controlled in the study and were administered in accordance with standard operating procedures in the PICU.

After PICU admission, the study was started when all examinations on admission were completed and all sources of irritation were eliminated. Electrodes were placed at F7, F8 and Fp (reference), and one electrode was placed beside Fp (ground) in accordance with the international ten-twenty system [29]. Electrode sites were abraded using conventional alcohol swabs. Paediatric silver–silver–chloride self-adhesive ECG electrodes were applied (Blue Sensor Neonatal™ Medicotest, Friedberg, Germany). An Aspect A-1000™ monitor (software version 3.12; Aspect Medical Systems, Natick, MA, USA) was used to compute the BIS. As recommended by the manufacturer, electrode impedances were kept below 10 kΩ, deviations among electrode impedances less than threefold, and signal quality index above 0.8.

### Group assignment

Patients were assigned *post hoc *into groups according to their level of sedation as measured using the COMFORT scale (deep sedation, light sedation, inadequate sedation) and into age groups (≤ 6 months and > 6 months of age). This age limit was used because in children younger than 6 months old [30] synchronization of the EEG is limited. Synchronization is among the columns of the BIS algorithm [11].

### Study protocol

**Figure 1:**
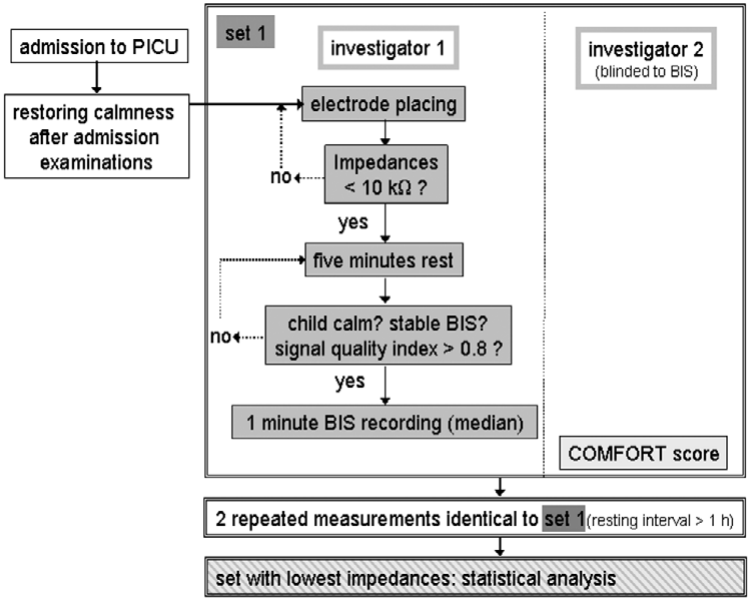
Study design. BIS, bispectral index; PICU, paediatric intensive care unit.

The study protocol is summarized in Fig. 1. After PICU admission, the first recording was started immediately after all necessary manipulations (examinations, laboratory specimens) were completed. If the data were of good quality (see above), then BIS values were sampled for 1 min and the median was calculated. A second investigator, who was blinded to the BIS results, assessed the patient clinically using the COMFORT scale [3]. Every patient was assessed three times, with a minimal interval of 1 hour between measurements. The set with the best impedance values was chosen for calculation of further statistics. To interpret the significance of the data quality, we also calculated the correlation between BIS and COMFORT score using the data couples with the poorest starting impedances. For each patient the following were also recorded: age, sex, medical diagnosis, Pediatric Risk of Mortality III score [31], medications administered, blood gases and temperature.

### Statistical analysis

Correlations between the BIS and the COMFORT score were calculated for each group using the Spearman's rank order correlation coefficient. Because BIS and COMFORT score were classified on an ordinal scale, regression analysis and confidence intervals could not be calculated. Therefore, we calculated the coefficient of determination (r^2^).

The ability of BIS to discriminate between sedation levels as classified using the COMFORT scale was tested using 'receiver operating characteristic' (ROC) statistics. The cut-off point was determined at the point of greatest sensitivity and specificity for discrimination. A logistic regression model using the BIS was developed to predict sedation levels of the patients (deep versus light sedation) in accordance with the COMFORT classification.

Data are expressed as mean ± standard deviation in the case of a normal distribution and interval scaling level, and as median (range) if the data were not distributed normally or in case of ordinal scaling level. The χ^2 ^and Fisher's exact tests were used to analyze categorical data and Mann–Whitney U-test for data on at least ordinal level. Data analysis was performed using SPSS statistical software (version 8.0; SPSS Inc., Chicago, IL, USA) and Microsoft Excel with Analyze It™ (version 1.48; Analyze-it Software Ltd, Leeds, UK) modification.

## Results

A total of 53 paediatric intensive care patients met our primary inclusion criteria. Three patients were excluded because of inability to achieve lead impedances with less than threefold deviance with the first set. In 10 patients recording could not be repeated at least twice because extubation was performed during the period of study. Finally, we enrolled 40 PICU patients into the study. According to COMFORT scoring no child was inadequately sedated, resulting in two COMFORT groups (i.e. deep sedation and light sedation). Patient characteristics are presented in Table 2. On comparing the sedation groups, we found that the parameters diagnosis (*P *= 0.162), Pediatric Risk of Mortality III score (*P *= 0.891), sex (*P *= 0.770) and age (*P *= 0.716) did not differ significantly between groups. We also found no significant differences in arterial carbon dioxide tension (*P *= 0.750), body temperature (*P *= 0.879) and type of medication (benzodiazepines, *P *> 0.99; opioids, *P *= 0.650; propofol, *P *= 0.286; ketamine, *P *> 0.99).

**Figure 2:**
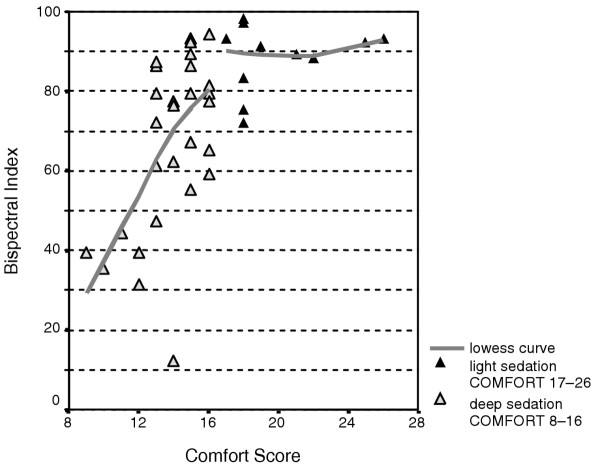
Correlation of bispectral index (BIS) and COMFORT score at different levels of sedation.

For those data couples with the best impedance levels (3.3 ± 1.8 kΩ, range 0.3–7.8 kΩ), BIS and COMFORT score correlated significantly for all patients (*n *= 40, *P *= 0.001; Spearman's rho: r = 0.651, r^2 ^= 0.42; Fig. 2) and for patients without ketamine (n = 38, *P *= 0.001; Spearman's rho: r = 0.668; r^2 ^= 0.45). The correlation between BIS and COMFORT for data couples with the worst impedances (5.1 ± 2.2 kΩ, range 1.9–9.9 kΩ) was poor (*P *= 0.05; Spearman's rho: r = 0.387, r^2 ^= 0.15). All further results reported are for the data couples with the best impedance levels.

Our results showed a significant correlation (*n *= 29, *P *= 0.003; Spearman's rho: r = 0.525, r^2 ^= 0.28) for deeply sedated patients (COMFORT score 8–16) and for patients who had not received ketamine (*n *= 27, *P *= 0.002; Spearman's rho: r = 0.565, r^2 ^= 0.32) whereas no correlation was found in the group with light sedation (COMFORT score 17–26; *n *= 11, *P *= 0.956; Spearman's rho: r = 0.019, r^2 ^< 0.01).

**Figure 3:**
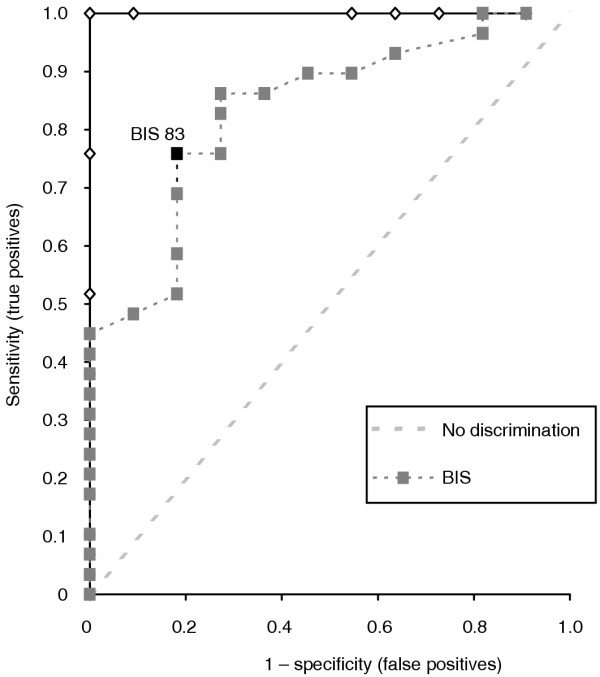
Receiver operating characteristic (ROC) analysis: cut-off point between light and deep sedation. BIS, bispectral index.

ROC analysis identified the BIS index level that distinguished best between deep and light sedation (groups classified by COMFORT scores). The calculated cut-off point between the groups was at a BIS of 83, which had a sensitivity of 75.9% and a specificity of 81.8% (area under the curve 0.834, 95% confidence interval 0.699–0.968; Fig. 3).

The logistic regression model using BIS as an explanatory variable was able to predict sedation according to COMFORT score correctly for 80% of the children. This total percentage is derived from correct predictions in 90% of deeply sedated patients (COMFORT score <17, *n *= 29) and in 55% of lightly sedated patients (COMFORT score 17–26, *n *= 11).

**Figure 4:**
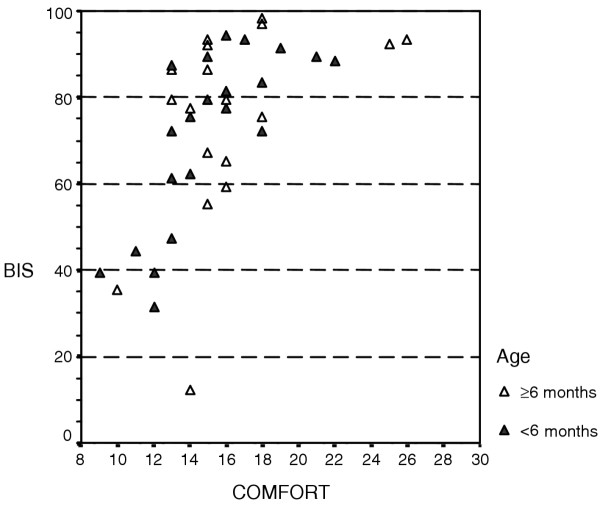
Correlation of bispectral index (BIS) and COMFORT score in patients younger and older than 6 months.

In patients younger than 6 months (*n *= 21, range 0.7–5.7 months) BIS and COMFORT score correlated significantly better (*P *< 0.001 in the younger group versus *P *= 0.041 in the older group; *n *= 19, range 6.3 months–16 years) and with a higher correlation coefficient (Spearman's rho: in younger patients r = 0.781, r^2 ^= 0.61 versus in older children r = 0.473, r^2 ^= 0.22; Fig. 4).

The correlation was slightly better in older children when the two patients who had received ketamine were excluded from the analysis (Spearman's rho: *n *= 17, *P *= 0.030; r = 0.527, r^2 ^= 0.28).

## Discussion

Most sedation scoring systems use responses to stimuli [32] and/or patient appearance and physiological variables [3] to estimate the level of sedation. These scores must be interpreted subjectively or, in case of physiological parameters, can be influenced by ICU therapy. In contrast to clinical scoring systems, the BIS system generates information continuously and objectively. The BIS monitor was developed to assess intraoperative depth of anaesthesia and to avoid awareness in adults. Data from studies that were not blinded sufficiently [25,26,28] or that employed scores that have not been evaluated in the PICU setting [25,27] suggested a moderate correlation between BIS and clinical scoring systems.

The aim of this prospective, blinded study was to determine whether BIS is a useful tool for assessing the level of sedation in critically ill paediatric patients. For statistical calculation only one data set (BIS versus COMFORT score) per patient was evaluated. Therefore, bias introduced by including multiple observations from one patient was avoided. In the study we compared BIS with the COMFORT score, which was previously validated in the PICU setting [3,4]. The study indicated that there was a moderate correlation between BIS and corresponding COMFORT scores (r^2 ^= 0.42). Subanalysis revealed a distinctly better coefficient of determination during deep sedation (r^2 ^= 0.28) than with light sedation (r^2 ^< 0.01) for the assessment tools. This was confirmed by the binary logistic regression findings, which revealed a good ability of BIS to predict deep levels of sedation (90%). According to COMFORT score, BIS could predict this level among lightly sedated children in only 55% of the cases. The overall percentage of correct prediction was 80%. Using ROC analysis we found a BIS value of 83 to distinguish best between light and deep sedation as assessed using the COMFORT score. Probably because of the exclusion of agitated children, we did not observe under-sedation in the study. We were therefore unable to differentiate further between lightly sedated and under-sedated children. This could be interpreted as an investigational bias. Furthermore, poor EEG data quality could cause a large number of movement artifacts, resulting in low signal quality, and we cannot exclude the possibility that movement artifacts contributed to the lower coefficient of determination for lighter sedated children than for children under deeper sedation. This was reported in adult settings [33,34].

Our data support the findings of previous studies [25-28]. Berkenbosch and colleagues [25] compared BIS with three simultaneously measured clinical sedation scores (Ramsay Sedation Score [RSS], Tracheal Suctioning Score, and Pediatric Intensive Care Unit Sedation Score) in paediatric patients (age 5.7 ± 6.1 years, range 1 month–20 years). None of the studied scores was clinically validated for use in paediatric ICU patients. The BIS monitor correlated moderately with clinically assessed sedation levels (r^2 ^= 0.12, 0.08 and 0.21, respectively). BIS was found to differentiate reliably between adequate and inadequate sedation (cut-off BIS 70), but it was relatively insensitive in differentiating between adequate and over-sedation (cut-off BIS 50). Critical aspects in the study conducted by Berkenbosch and coworkers are that different individuals performed the sedation assessments, which might have resulted in considerable interobserver variability. The nurses assessing the level of sedation were not formally blinded to the BIS results. Multiple sets of data derived from single patients were included in same analysis, which might have influenced the results. Twenty-four patients were included in the study, but measurements were repeated 18 ± 14 times per patient.

Crain and colleagues [26] also used the COMFORT score to estimate the level of sedation. Those investigators studied 31 patients (age 53 ± 11 months, median 25 months; no range presented) and selected each patient's lowest and highest BIS measurement for further investigation. The direct coefficient of determination between BIS and COMFORT score was only moderate (r^2 ^= 0.26), which reflected their finding that some patients exhibited good correlation whereas others did not. After grouping BIS results into four levels of sedation, a high coefficient of determination (r^2 ^= 0.89) with the COMFORT score resulted. Grouping of BIS values was conducted according to results formerly derived from adult data, but it is not proven whether this procedure is appropriate in children.

Aneja and coworkers [27] studied 24 patients without neuromuscular blockade, comparing BIS with RSS. They found a high and significant coefficient of determination (r^2 ^= 0.77; age 6.3 ± 2.9 years, range 1–16 years). The calculated cut-off point for distinguishing between over-sedation (RSS 6) and comfortable sedation (RSS 2–5) by ROC analysis was a BIS level of 42. Under-sedation (RSS 1) was found at a BIS level in excess of 76. Another component of that study dealt with patients receiving neuromuscular blockade (age 8.4 ± 3.7 years, range 0.5–19 years). According to BIS, the authors observed a significant number of patients suffering from inadequate sedation, which would not have been detectable by clinical investigation. Limitations of the study were the inclusion of multiple observations per patient and that the RSS has not been validated for use in paediatric patients, as mentioned above.

Courtman and coworkers [28] recently compared BIS and COMFORT score in critically ill children (mean age 3.9 ± 4.5 years). Those investigators found a moderate coefficient of determination (r^2 ^= 0.26) between BIS and COMFORT score in 25 neurologically normal children and a weak coefficient of determination (r^2 ^= 0.06) in 15 children who were classified as being neurologically abnormal. In that study BIS could discriminate between light and deep levels of sedation. In conformance with the findings of Berkenbosch and coworkers [25], Courtman and colleagues found that BIS was unable to discriminate between deep and very deep levels of sedation. The significance of this study is also limited by the inclusion of multiple observations per patient.

In our study the quality of EEG impedance appeared to have a major impact on correlation between BIS and COMFORT score. Although within the limits recommended by the manufacturer, we found only a low coefficient of determination (r^2 ^= 0.15) between COMFORT score and corresponding BIS in case of higher impedances (5.1 ± 2.2 kΩ). This emphasizes the importance of good data quality. On the basis of our experience, we would advise use of impedance values of less than 5 kΩ.

Unexpectedly, correlation between the methods was better in children younger than 6 months. Patient basic data do not explain this finding. The impact of age on BIS is still debated, with divergent findings reported in the anaesthesia literature [21,35]. Davidson and coworkers [35] compared BIS with the corresponding consciousness level during emergence from anaesthesia in a prospective, blinded manner in children (≥1 year old) and infants (<1 year old) undergoing elective circumcision. BIS increased significantly as sevoflurane concentrations decreased in children, but a similar relationship was not demonstrated in infants. Adult EEG sensors were used in that study for all patients, which could partly account for the difference in findings between that study and ours. There are no additional data in the paediatric ICU literature.

Because different scoring systems are used for clinical estimation of the level of sedation, it is difficult to compare our findings with those of other investigators. The end-points of sedation are neither defined consistently nor comparable between the studies mentioned above. In our study COMFORT scores corresponded to a wide range of BIS values. In other words, the BIS index does not always reflect the expected clinical/subjective level of sedation. This observation is in agreement with the experience of other groups who compared neurophysiological parameters (i.e. BIS, somatosensory evoked magnetic fields, evoked potentials) with clinical sedation scores in adult patients [36-38] and with the other paediatric ICU studies [25-28]. This could be related to the fact that neurophysiological parameters and clinical sedation scores measure different attributes. BIS automatically matches certain EEG patterns to clinical states that are found in adult volunteers under sedation or anaesthesia. In contrast, clinical scores are used to summarize the investigator's impression of whether the patient is comfortable in the ICU setting. An extreme example would be the patient who is awake with a high BIS score but who appears to be completely comfortable. The limitations of clinical scores in estimating the level of sedation have been discussed broadly [32]. In addition to these limitations, in the case of the COMFORT score there is a specific limitation caused by the inclusion of physiological data (heart rate and blood pressure). When physiological parameters are used in a heterogeneous population such as paediatric ICU patients, it is difficult to define reference data. If changes in physiological parameters caused by sedatives are to be interpreted, then a standardized sedation regimen and co-medication are needed. In our opinion this is not the case in the ICU setting. The majority of children included in the present study were postoperative cardiac surgical patients. Most of them had reduced cardiac function and required catecholamines. In this group of patients in particular, haemodynamic variability could not be attributed solely to sedative drugs.

The COMFORT score cannot distinguish between very deep stages of sedation, whereas with neurophysiological methods such as the BIS this might be possible. EEG measurements often fail to provide correct values with very light or absent sedation because motor activity and skeletal muscle tone increase. However, this situation will never fall within the domain of electrophysiological monitoring because clinical estimation will be sufficient in unsedated or only lightly sedated patients in the ICU setting.

Data from the investigations cited above and our data suggest that the BIS monitor might be useful in the case of moderate to deep levels of sedation. Our group found that BIS levels below 83 had good correlation with clinical estimation. Berkenbosch and coworkers [25] suggested that a moderate level of sedation could be achieved at BIS values between 50 and 70, and deep sedation at levels below 50. This corresponds well with the data presented by Aneja and coworkers [27], who found that a BIS above 76 indicated inadequate, light sedation and that a BIS below 46 marked very deep sedation or over-sedation. Whether BIS can detect over-sedation remains controversial. Berkenbosch and coworkers [25] and Courtman and colleagues [28] found that BIS could not discriminate between deep and very deep levels of sedation. In our opinion, this finding could indicate that clinical scores are not useful for identifying very deep levels of sedation.

Few data exist for paralyzed PICU patients. In this situation clinical scores are not applicable because they need muscular activity to be present to rate the level of sedation. Aneja and coworkers [27] stated that the RSS and bedside nurse assessments are inadequate for monitoring the depth of sedation in paralyzed children, and concluded that BIS is a useful adjunct for assessing sedation in paralyzed patients. In our opinion use of electrophysiological monitoring tools such as the BIS is imperative in paralyzed patients to prevent levels of sedation that are too light or too deep.

Furthermore, the terms 'under-sedation' and 'over-sedation' should be used carefully because the level of sedation required depends on the individual patient's needs. Bearing this in mind, clinician should adapt the level of sedation to the demand of the patient. When deeper levels of sedation are needed, BIS can help by avoiding undesired levels of sedation. In situations when muscular paralysis is necessary, BIS could make a valuable contribution because established clinical tools fail to measure the level of sedation in this setting.

## Conclusion

Our data indicate that an impedance level below 5 kΩ is needed for valid interpretation of BIS values in PICUs. At such impedance levels the BIS index correlates well with the COMFORT sedation score, in particular in children with deeper levels of sedation. Discrimination between light and moderate sedation with high sensitivity and specificity was possible at a BIS level of 83. The BIS monitor provides continuous measurements of the level of sedation without having an impact on patient comfort. It may be a useful adjunct in clinical routine and may be especially helpful in certain situations when clinical estimation fails (e.g. if muscular paralysis is necessary).

## Key messages

• 	In daily clinical practice of paediatric ICU therapy, the BIS monitor provides continuous measurements of sedation level without having an impact on the patient's comfort.

• 	In certain situations when clinical estimations fail (i.e. if muscular paralysis is necessary), electrophysiological monitoring tools such as BIS should be considered imperative to prevent inadequately light or inadequately deep sedation.

• 	In the case of deep sedation, BIS correlated satisfactory with the COMFORT score results if low EEG impedances were guaranteed.

## Abbreviations

BIS = bispectral index; EEG = electroencephalography; ICU = intensive care unit; PICU = paediatric intensive care unit; ROC = receiver operating characteristic; RSS = Ramsay Sedation Score.

## Competing interests

The author(s) declare that they have no competing interests.

## Authors' contributions

All authors contributed to the design, conduct, analysis and interpretation of the research reported. CS and WK were principal investigators and led the conceptual design of the study. MS and GN assisted with data collection and analysis, and with manuscript preparation.

## Figures and Tables

**Table 1:** The COMFORT scale

Alertness	Calmness/agitation	Respirator response	Physical movement	Blood pressure (MAP)	Heart rate	Muscle tone	Facial expression	Points
Deeply asleep	Calm	No coughing and no spontaneous respiration	No movement	Below baseline	Below baseline	Totally relaxed; no tone	Totally relaxed	1
Lightly asleep	Slightly anxious	Spontaneous respiration with little or no response to ventilation	Occasional, slight movement	Consistently at baseline	Consistently at baseline	Reduced	Normal; no facial tension evident	2
Drowsy	Anxious	Occasional cough or resistance to ventilator	Frequent, slight movement	Infrequent elevations of 15% or more (1–3/observ.)	Infrequent elevations of 15% or more (1–3)	Normal	Tension evident in some facial muscles	3
Fully awake and alert	Very anxious	Actively breathes against respirator or coughs regularly	Vigorous movement limited to extremities	Frequent elevations of 15% or more (>3/observ.)	Frequent elevations of 15% or more (>3)	Increase tone and flexion of fingers and toes	Tension evidence throughout facial muscles	4
Hyper alert	Panicky	Fights ventilator, coughing or choking	Vigorous movement, including torso and head	Sustained elevation ≥15%	Sustained elevation ≥15%	Extreme muscle rigidity and flexion of fingers and toes	Facial muscles contorting and gromacing	5

MAP, mean arterial pressure. Data from Ambuel and coworkers [3].

**Table 2:** Patient characteristics

Characteristic/parameter	All patients	Deeply sedated	Lightly sedated	*P*
Number	40	29	11	-

Age (median [range])	5.6 months (21 days–16 years)	5.7 months (1.5 months–13 years)	5.1 months (21 days–16 years)	0.716
Sex (*n*[%])				
Male	21 (52)	16 (55)	5 (45)	0.770
Female	19 (47)	13 (45)	6 (55)	
Diagnosis (*n*[%])				
Cardiac	34 (85)	23 (79)	11 (100)	0.162
Gastrointestinal	2 (5)	2 (7)	0 (0)	
Other	4 (10)	4 (14)	0 (0)	
PRISM III score (mean ± SD)	7.22 ± 5.29	7.34 ± 5.78	6.91 ± 3.96	0.891
Medication (*n*[%])				
Benzodiazepines	34 (85)	25 (86)	9 (82)	≥ 0.999
Opioids	33 (83)	23 (79)	10 (91)	0.650
Propofol	13 (32)	11 (38)	2 (18)	0.286
Ketamine	2 (5)	2 (7)	0 (0)	≥ 0.999
PaCO_2 _(kPa; mean ± SD)	5.1 ± 0.8	5.1 ± 0.9	4.9 ± 0.8	0.750
Temperature (°C; mean ± SD)	37.4 ± 0.7	37.4 ± 0.6	37.4 ± 0.7	0.879

*P *values are given for the comparison of deeply sedated versus lightly sedated. PRISM, Pediatric Risk of Mortality III score, PaCO_2_, arterial carbon dioxide tension; SD, standard deviation;
