# Correction: Bispectral index versus COMFORT score to determine the level of sedation in paediatric intensive care unit patients: a prospective study

**DOI:** 10.1186/cc3810

**Published:** 2005-09-02

**Authors:** Andreas E Triltsch, Grit Nestmann, Helmut Orawa, Maryam Moshirzadeh, Michael Sander, Joachim Große, Arka Genähr, Wolfgang Konertz, Claudia D Spies

**Affiliations:** 1 Department of Anesthesiology and Intensive Care Medicine, Campus Benjamin Franklin, Charité University Hospital Berlin, Berlin, Germany; 2 Department of Pediatrics, Campus Benjamin Franklin, Charité University Hospital Berlin, Berlin, Germany; 3 Department of Medical Informatics, Biometry and Epidemiology, Charité University Medicine Berlin, Berlin, Germany; 4 Department of Anesthesiology and Intensive Care Medicine, Campus Charité Mitte, Charité University Hospital Berlin, Berlin, Germany; 5 Department of Cardiac Surgery, Campus Charité Mitte, Charité University Hospital Berlin, Berlin, Germany

After the publication of this article [1] we noticed that the affiliation details were incorrect and should be as follows:

Andreas E Triltsch – Department of Anesthesiology and Intensive Care Medicine, Campus Benjamin Franklin, Charité University Hospital Berlin, Berlin, Germany

Grit Nestmann – Department of Pediatrics, Campus Benjamin Franklin, Charité University Hospital Berlin, Berlin, Germany

Helmut Orawa – Department of Medical Informatics, Biometry, and Epidemiology, Charité University Medicine Berlin, Berlin, Germany

Maryam Moshirzadeh, Michael Sander, Joachim Große and Claudia D Spies – Department of Anesthesiology and Intensive Care Medicine, Campus Charité Mitte, Charité University Hospital Berlin, Berlin, Germany

Wolfgang Konertz – Department of Cardiac Surgery, Campus Charité Mitte, Charité University Hospital Berlin, Berlin, Germany
